# The MIND Diet and Physical Activity in Cognitive Ageing: From Mechanisms to Multidomain Trials

**DOI:** 10.3390/nu18213579

**Published:** 2026-10-29

**Authors:** Yu Liang Lim, Xuanmin Tan, Li Cheng Heng, Chin Yee Cheong

**Affiliations:** 1 Department of Geriatric Medicine, Khoo Teck Puat Hospital, Singapore 768828, Singapore; 2 Lee Kong Chian School of Medicine, Nanyang Technological University, Singapore 308232, Singapore

**Keywords:** MIND diet, physical activity, multidomain intervention, cognitive preservation

## Abstract

Preserving cognitive function against the effects of ageing and neurodegenerative disease is the goal of lifestyle-based approaches to brain health. This review examines the evidence for the Mediterranean-DASH Intervention for Neurodegenerative Delay (MIND) diet, for physical activity covering aerobic, resistance, and mind–body exercise modalities, and for the multidomain programmes in which both are embedded. For each, we consider the mechanistic rationale, the biomarker, neuroimaging, and the observational and interventional evidence. Overall, a substantial body of evidence links both exposures to better cognitive performance; however, uncertainties remain regarding optimal intervention dose, duration, target populations, and whether MIND diet and exercise exert independent or synergistic effects. We further propose that the discordance between cohort and trial findings may reflect baseline neurodegenerative risk, with benefit being hardest to detect in low-risk populations over short follow-up. Few trials have tested the MIND dietary pattern change alongside structured exercise, and none has isolated the contribution of either within a multidomain programme. More strategically designed trials, with clinical endpoints and single domain comparators, are needed to answer these questions.

## 1. Introduction

Population ageing has driven a rise in neurodegenerative disorders. An estimated 57.4 million individuals worldwide were living with dementia, a figure projected to reach 152.8 million by 2050 [1], with a staggering global societal cost of dementia estimated to be US$1.3 trillion in 2019, with approximately 50% attributable to informal caregiving [2,3]. In the absence of curative pharmacotherapies, the attention has shifted toward prevention and early intervention. The 2024 Lancet Commission on Dementia Prevention estimated that up to 45% of dementia cases may be attributable to modifiable risk factors. Several of these include hypertension, obesity, physical inactivity, diabetes, high LDL, etc. [4]. These factors are modified directly by diet and exercise, placing lifestyle intervention among the few prevention strategies available at population scale.

This review focuses on two accessible and scalable lifestyle strategies: the Mediterranean-DASH Intervention for Neurodegenerative Delay (MIND) diet and physical activity. The rationale for considering them together rests on more than their independent efficacy. A substantial body of mechanistic evidence indicates that diet and exercise converge on the same core neurobiological substrates such as cerebrovascular and endothelial function, neuroinflammation and oxidative stress, and neurotrophic signalling, and it is believed that their combined application may exert complementary effects on synaptic plasticity and cognition [5]. They are also the two components common to every major multidomain prevention trial conducted to date, yet they are more often reviewed in isolation than together, and the question of whether their combination confers synergistic benefit beyond either alone remains largely unknown. Prospective cohorts report substantial protective associations for MIND diet adherence [6,7], while the largest randomised trial of the diet was null [8]; multidomain interventions have produced clear cognitive benefit in some populations and none in others, sometimes within the same trial network and under closely comparable protocols [9,10,11,12,13]. This discordance is usually attributed to methodological heterogeneity. An additional hypothesis is that baseline cognitive and neurodegenerative risk may modify the observable benefits of lifestyle intervention, with effects potentially less detectable in low-risk populations due to limited cognitive decline during follow-up, and in those with advanced neuropathology due to reduced capacity for modification. However, this proposed relationship remains speculative and requires prospective validation. Accordingly, this narrative review synthesises mechanistic, biomarker, observational and interventional evidence for the MIND diet and physical activity independently, before examining how these two lifestyle cornerstones are incorporated within multidomain intervention strategies, and considers the implications of the evidence for clinical translation.

### Literature Search and Selection

A structured literature search was conducted in PubMed/MEDLINE, Embase, and Scopus from database inception to August 2026. Search terms included “MIND diet,” “Mediterranean diet,” “physical activity,” “exercise,” “aerobic exercise,” “resistance training,” “cognition,” “cognitive decline,” “mild cognitive impairment,” and “dementia,” combined using Boolean operators. Eligible publications included observational studies, randomised controlled trials, systematic reviews, and meta-analyses examining dietary patterns and/or physical activity in relation to cognitive outcomes. Relevant mechanistic, biomarker, and neuroimaging studies were also considered. English-language publications were selected through title, abstract, and full-text screening based on their relevance to the review objectives. Evidence was synthesised qualitatively across mechanistic, observational, and interventional domains.

## 2. The MIND Diet

### 2.1. Composition and Scoring

The MIND diet was introduced in 2015 by Morris et al. as a hybrid of the Mediterranean and Dietary Approaches to Stop Hypertension (DASH) diets, specifically designed to target the neuropathological processes underlying cognitive decline and dementia [14]. It comprises 15 dietary components classified into 10 “brain healthy” food groups to be encouraged, and five food groups to be limited. Each component is scored 0, 0.5 or 1 according to intake frequency, giving a total of 0 to 15; the half-point tier captures partial adherence and accounts for the non-integer scores reported through the cohort literature [14]. Two scoring features merit comment. First, the wine component awards a full point only for intake of one glass daily, with abstention and two or more glasses both scoring zero. Its inclusion is now difficult to justify, and several adaptations have removed or substituted it [15]. Importantly, this scoring criterion should not be interpreted as a recommendation to initiate wine consumption for cognitive protection. Second, absolute scores are not strictly comparable across studies, given differing dietary instruments, serving size conversions, inconsistently reported scoring decisions [16], and culturally adapted components [15].

### 2.2. Mechanisms of Neuroprotection

The cognitive benefits of the MIND diet have been attributed to multi-targeted neurobiological action. Experimental evidence suggests that these constituent bioactive compounds such as antioxidant vitamins (E, C), carotenoids, polyphenols, flavonoids, B vitamins (B6, B9, B12), omega-3 polyunsaturated fatty acids (PUFAs), monounsaturated fatty acids (MUFAs), dietary fibre, and phylloquinone are proposed to act in concert across four principal mechanistic axes: amyloid proteostasis, neuroinflammation and oxidative stress, vascular integrity, and synaptic plasticity [14]. However, much of this mechanistic evidence is derived from animal models or individual nutrients, and direct evidence demonstrating equivalent effects from the complete MIND dietary pattern in humans remains limited. The key bioactive compounds and their proposed mechanistic pathways across individual MIND diet components are summarised in Table 1.

#### 2.2.1. Amyloid Proteostasis

In experimental models, dietary antioxidants [17], B vitamins [18], polyphenols, and PUFAs [19] have been shown to inhibit the formation of amyloid-beta (Aβ) oligomers and fibres. Oleocanthal, the key phenolic compound of extra virgin olive oil (EVOO), enhanced Aβ clearance by increasing the activity of Aβ transport proteins at the blood–brain barrier (BBB) [20,21]. In rodent studies, omega-3 PUFAs (DHA and EPA) from fish promote efficiency of the glymphatic system enhancing interstitial Aβ clearance via an Aquaporin-4 (AQP4)-dependent pathway [22], although the exact clinical relevance of this mechanism in humans remains uncertain. Simultaneously, exogenous antioxidants with a focus on vitamin E from nuts and whole grains may exert anti-inflammatory effects and modulate oxidative stress through cellular signalling pathways and endogenous antioxidant defences, thereby reducing the oxidative damage induced by β amyloid, that would otherwise induce cytotoxicity [23].

#### 2.2.2. Neuroinflammation and Oxidative Stress

Chronic neuroinflammation is an established driver of neurodegeneration [24]. Omega-3 fatty acids suppress pro-inflammatory cytokine production by inhibiting nuclear factor-κB (NF-κB) activation, which is a key transcription factor involved in the expression of genes encoding proteins involved in inflammation, and generating specialised pro-resolving mediators (resolvins, protectins) [25], while dietary polyphenols commonly found in many of the plants produce similarly block-NF-κB-dependent inflammatory gene expression [26]. The MIND diet’s broad antioxidant provision, encompassing vitamins E and C, and carotenoids from leafy greens and nuts, limits the generation of lipid peroxidation products, which have been implicated in amyloid β misfolding and synaptic dysfunction [27]. Meanwhile, MUFAs abundant in olive oil appear to have anti-inflammatory effects that may attenuate the chronic inflammation of inflammatory cytokines such as TNF-α and IL-6, which are implicated in dementia risk [28]. Adherence to the MIND diet has been associated with lower odds of hippocampal sclerosis and with attenuated inflammation cognition relationships [29,30].

#### 2.2.3. Preservation of Vascular Cerebral Health

Individual components of the MIND diet synergistically promote vascular health through improvements in lipid profile, glucose metabolism, and anthropometric indices. Adherence to a Mediterranean diet lowers oxidised low-density lipoprotein, which is a promoter of oxidative stress-associated neurodegenerative disorders [31], improves glucose control [32], and promotes weight loss [33]. These cardiometabolic improvements optimise vascular cerebral health by preserving endothelial function, reducing oxidative stress and atherosclerotic burden, and limiting disruption of the blood–brain barrier [34]. Improved cerebral blood flow in turn attenuates neurotoxic accumulation, minimising neuronal dysfunction. In addition, it improves amyloid-β clearance at the blood–brain barrier, preventing hyperphosphorylation of tau, which leads to neurofibrillary tangle formation [35].

#### 2.2.4. Neurotrophic Signalling and Synaptic Plasticity

Anthocyanins and other flavonoids, which are particularly abundant in berries, upregulate BDNF expression in experimental models, promoting synaptic plasticity and adult hippocampal neurogenesis [36,37]. This axis is of particular relevance to integration developed in Section 3, since BDNF-mediated plasticity is also a principal pathway through which aerobic exercise is thought to act.

**Table 1:** Individual MIND diet components: Key bioactives and mechanistic pathways ^A^.

Component	Key Bioactives	Potential Mechanistic Pathway(s)
Whole Grains [38,39]	Fibre, polyphenols, B vitamins, vitamin E, complex carbohydrates	Glycaemic stabilisation minimising insulin resistance; modulation of gut microbiota for improvement of gut–brain axis with SCFA production
Green Leafy Vegetables [40] (kale, collards, greens; spinach; lettuce/tossed salad)	Lutein, phylloquinone (Vit K1), folate, α-tocopherol, nitrate, kaempferol	Potential neuroprotective effects of micronutrients: Carotenoids → associated with less severe periventricular white matter lesions; Lutein → may reduce phospholipid peroxidation and attenuate oxidative stress, mitochondrial dysfunction, and neuroinflammation; Folate → may inhibit tau phosphorylation and reduce APP, PS1, and Aβ protein levels
Berries [41]	Anthocyanins, flavonoids, polyphenols	Antioxidant properties, influencing cell signalling cascade helping to reduce age-related declines in neuronal signalling and reductions in neuroinflammation; BDNF upregulation promoting synaptic plasticity and neurogenesis
Fish [42] (tuna sandwich, fresh fish as main dish, not fried)	Omega-3 PUFAs (DHA, EPA)	Long-chain n-3 fatty acids may reduce Aβ formation and oxidative damage while increasing synaptic protein levels and dendritic spine density
Olive Oil [43] (as main primary oil)	Oleocanthal, polyphenols, MUFAs	Rich in phenolic compounds, which are believed to have neuroprotective effects; enhanced Aβ clearance; neutralises the cytotoxicity of accumulated Aβ and reduces inflammation from activated astrocytes and microglia
Nuts [44]	Vitamin E, fatty acids, polyphenols	Micronutrients help improve endothelial function and cardiovascular health, and provide anti-inflammatory effects by reducing oxidative stress
Beans (beans, lentils, soybeans)	Fibre, folate, complex carbohydrates	Reduce reactive oxygen species; ameliorate β-amyloid-induced cellular apoptosis; source of folate which downregulates homocysteine
Poultry (chicken or turkey, not fried)	Protein	Aims to be a dietary and protein substitution for neuroinflammatory red meat components
Wine	Polyphenols	Antioxidant and anti-inflammatory agent, reduces tau phosphorylation, reduces Aβ-induced production of reactive oxygen species [45]. Wine intake should be limited to moderate consumption, consistent with the MIND diet scoring system
Restricted items (red meat, butter and margarine, cheese, pastries and sweets, and fried or fast food)	Saturated fats, trans fats, cholesterol, dAGEs, high salt content	Saturated and trans fats lead to blood–brain barrier dysfunction and increased Aβ aggregation [46]; reduce BDNF; increase insulin resistance, resulting in accumulation of β amyloids; dAGE induces oxidative stress, pro-inflammatory, increases insulin resistance and lipid peroxidation, all resulting in neurotoxicity [47]

Abbreviations: dAGE = dietary advanced glycation end product; BDNF = brain-derived neurotrophic factor; DHA = docosahexaenoic acid; EPA = eicosapentaenoic acid; MUFA = monounsaturated fatty acid; PUFA = polyunsaturated fatty acid; and SCFA = short-chain fatty acid. ^A^ The mechanistic pathways summarised are largely derived from experimental and nutrient-level evidence and should not be interpreted as established effects of the complete MIND dietary pattern.

### 2.3. Biomarker Evidence, Neuroimaging, and Neuropathological Studies

Biomarker, neuroimaging, and neuropathological studies provide growing biological support for the neuroprotective potential of the MIND diet, although the strength of evidence varies across pathological domains. At the amyloid neuropathological level, in the Rush Memory and Aging Project [48], MIND diet adherence was associated with lower global AD pathology and reduced β-amyloid burden on postmortem examination, with each one-point increase in MIND score corresponding to an amyloid-related pathological burden approximately equivalent to 4.25 fewer years of age. In contrast, MIND diet adherence was not significantly associated with p-tau tangle burden, suggesting that its neuropathological associations may be more closely related to amyloid-related pathways than to tau pathology. This pattern is also consistent with the mechanistic evidence discussed in Section 2.2, in which several components of the MIND diet have been implicated in Aβ formation, clearance, and proteostasis.

This amyloid signal is also supported by biomarker and neuroimaging studies. In the ALBION study, higher MIND adherence was associated with lower odds of pathological CSF Aβ_42_ concentrations in cognitively unimpaired middle-aged adults [49]. Similar findings have been reported with Mediterranean-style dietary patterns, which form the dietary foundation of the MIND diet. Higher Mediterranean or MIND diet adherence has been associated with lower cerebral amyloid deposition on ^11^C-PiB PET [50], while longitudinal studies suggest that lower adherence may be accompanied by greater accumulation of amyloids and faster cerebral hypometabolism over time [51,52]. Collectively, these findings provide convergent evidence across postmortem tissue, CSF biomarkers, and PET imaging that greater adherence to MIND or closely related Mediterranean dietary patterns may be associated with a lower amyloid burden.

Evidence for the preservation of brain tissue structure is less consistent. Observational MRI studies have generally suggested that healthier dietary adherence may be associated with greater preservation of total brain or hippocampal volume, although associations with cortical thickness, grey matter volume, and other structural measures have not been uniformly demonstrated [51,52,53]. These findings suggest that the potential biological effects of dietary adherence may extend beyond amyloid metabolism to broader structural pathways. However, the interventional evidence remains considerably less convincing. The neuroimaging sub-study of a landmark RCT–Trial of the MIND Diet for Prevention of Cognitive Decline in Older Persons showed no significant between-group differences in white matter hyperintensity volume, hippocampal volume, or total grey and white matter volumes over three years between the MIND diet and the caloric restrictive control group [8]. Interpretation is limited by the small imaging subsample, and using an active control diet that also incorporated caloric restriction and dietary improvement, which may have reduced the biological contrast between groups.

Taken together, the biomarker literature provides a consistent support for an association between MIND or similar Mediterranean-style dietary adherence and lower amyloid burden, with more tentative evidence for favourable effects on structural brain change. However, these associations do not establish causality or demonstrate that dietary intervention directly modifies amyloid production or clearance in humans, particularly as randomised biomarker and neuroimaging evidence is still limited. Nevertheless, these findings provide a biological rationale for the MIND diet as a neuroprotective strategy, while underscoring the need for adequately powered, long-duration trials with integrated biomarker and radiological endpoints to confirm the magnitude and durability of these effects.

### 2.4. Key Clinical Evidence: Observational and Interventional Studies

Building upon this mechanistic and biomarker foundation, observational and interventional studies have examined whether adherence to the MIND diet translates into meaningful reductions in cognitive decline and dementia risk across diverse adult populations.

The seminal evidence was established [14] in the Memory and Ageing Project (MAP), in which 960 Chicago older adults without dementia were followed over 10 years. MIND diet adherence was associated with slower cognitive decline, with participants in the highest adherence tertile estimated to be cognitively 7.5 years younger than those in the lowest. Benefit was also observed with moderate adherence, and associations persisted after adjustment for cardiovascular risk factors, suggesting that the relationship may not be explained solely by cardiovascular risk reduction. In a separate analysis of 923 participants from the same MAP cohort, Morris et al. [6] found that higher MIND diet adherence was associated with lower incidence of Alzheimer’s disease over a mean follow-up of 4.5 years, and the observation persisted after adjustment for cardiovascular risk factors and other potential confounding factors.

However, the strength of this association has not been uniformed across all cognitively unimpaired populations. In the Nurses’ Health Study, a large prospective cohort of 16,058 American women aged ≥70 years, long-term MIND diet adherence was associated with better verbal memory but not with global cognitive decline over six years [54]. Likewise, the DO-HEALTH study found no significant association between baseline MIND adherence and cognitive decline over three years in 2028 generally healthy community-dwelling older adults across five European countries [55]. The same pattern was seen in the Framingham Heart Study (FHS) cohort, where higher MIND adherence was associated cross-sectionally with better global cognition and larger total brain volume, but not with cognitive decline over a mean 6.6 years of follow-up [56]. What these three cohorts share is comparatively low baseline vulnerability. All were free of dementia and largely free of clinically significant cardiovascular disease at entry, and significantly younger at baseline than the MAP participants. In relatively healthy populations with low short-term risk of decline, fewer cognitive events and smaller changes over follow-up may reduce the ability to detect an effect. This supports the possibility that the benefits of MIND adherence are most readily observable in populations with sufficient underlying neurodegenerative or vascular risk for cognitive trajectories to diverge measurably over time.

Additionally, sub-analysis of the Nurses’ Health study [54] also further demonstrated that excluding the unhealthy components, namely butter and margarine, as well as pastries and sweets, attenuated its protective association with verbal memory, underscoring that the diet’s cognitive benefits are dependent not only on adequate intake of its 10 brain healthy food groups, but also on adherence to the restriction of its five unhealthy components, which together define the diet’s full neuroprotective profile.

Longer-term cohort evidence provides additional support for MIND diet efficacy. In the Australian PATH Through Life cohort study of 1220 cognitively unimpaired adults with mean age 62 years, higher MIND adherence was associated with substantially lower 12-year odds of incident cognitive impairment, with each one-point increase in MIND score associated with a 19% reduction in odds [7]. Importantly, cognitive impairment was adjudicated using neuropsychological testing, MMSE performance, and functional assessment rather than defined solely by changes in a composite cognitive score. This suggests that the association may extend beyond test score differences to clinically meaningful transitions from normal cognition to MCI or even dementia.

The potential applicability of the MIND framework beyond Western dietary settings has also been explored. In the China Health and Nutrition Survey of 4066 Chinese individuals with mean age of 62.2 years, a culturally adapted 12-component MIND diet scoring foods was developed to reflect local dietary habits. The adaption excluded olive oil, butter and margarine, and cheese, which were rarely consumed in this population, while replacing wine with tea, given their shared polyphenol-rich composition. Despite these modifications, higher adherence remained associated with better global cognitive function, with every three-point increase in score corresponding to an effect size approximately equivalent to one year of younger cognitive age over a median study follow-up of 3 years [57]. These findings suggest that the neuroprotective framework of the MIND diet retains its cognitive relevance across widely divergent dietary contexts and cultural populations, provided its core mechanistic principles are preserved even when specific food items are substituted.

Evidence from a recent landmark RCT, however, is more equivocal. The largest randomised trial to date on the MIND diet is the Trial of the MIND Diet for Prevention of Cognitive Decline in Older Persons, which enrolled cognitively unimpaired older adults with a family history of dementia, BMI ≥ 25 kg/m^2^, and a suboptimal diet defined by a baseline MIND score of ≤8, randomising them to either a MIND diet with mild caloric restriction or an active control diet with equivalent caloric restriction. Global cognition over three years improved in both groups, with no statistically significant between-group difference (SMD 0.035, 95% CI −0.022–0.092) [8]. Interpretation is complicated by the control intervention. Both groups received caloric restrictions and lost weight. By trial completion, mean MIND diet scores had increased to approximately 11.0 in the intervention group and 8.5 in the control group, where the control group also exceeded the median score associated with cognitive protection in the middle adherence tertile of the original MAP cohort. The between-group dietary contrast was therefore narrower than intended, making this a comparison between greater and lesser degrees of dietary improvement, rather than between a MIND diet and an unchanged habitual diet. Without an untreated comparator, the improvement seen in both groups also cannot be attributed specifically to diet, since weight loss and repeated cognitive testing remain plausible contributors too.

Higher MIND diet scores have generally been associated with better cognitive outcomes, but no threshold for benefit can be identified. The absolute scores at which associations appear vary widely. In MAP, both moderate and higher adherences were associated with lower Alzheimer disease risk, with median scores of 7.5 and 9.5 respectively, compared with 6 in the lowest tertile. In the PATH Through Life cohort, each one-point increase in MIND score was associated with lower odds of incident cognitive impairment, while the highest adherence group had a mean score of only 7.8. By contrast, in the culturally adapted Chinese cohort, favourable cognitive associations were observed at substantially lower absolute scores, with a median score of 4.5 out of 12 and a mean of 5.5 in the highest tertile. Taken together, these findings suggest that greater adherence generally produces favourable results, but no single score can be considered an efficacy threshold. Differences in dietary assessment, scoring adaptations, population, follow-up, and outcome definitions further limit direct comparisons across studies. MIND scores should therefore be interpreted within the context of each study.

Overall, the current evidence is supportive of an association between greater MIND diet adherence and more favourable cognitive outcomes, particularly over longer follow-up and in populations with sufficient underlying risk for cognitive decline to become detectable. The strongest support comes from prospective cohorts, where higher adherence has been associated with slower cognitive decline and lower incidence of cognitive impairment. However, findings are less consistent in healthier, lower-risk populations, and randomised evidence remains limited. Further long-duration randomised trials with clearer dietary separation between intervention and control groups are needed to determine the magnitude of any cognitive benefit. The key observational and interventional studies examining the association between MIND diet adherence and cognitive outcomes are summarised in Table 2.

## 3. Physical Activity

### 3.1. Mechanistic Pathways Linking Physical Activity and Cognition

Physical inactivity is among the modifiable risk factors identified by the Lancet Commission. The literature conventionally divides physical activity into three common modalities: aerobic exercise (sustained cardiovascular loading), resistance training (progressive mechanical loading of skeletal muscle), and mind–body exercise (coordinated movement integrated with sustained attentional focus). Though each modality converges on several shared mechanistic pathways, each additionally recruits pathways distinct to themselves.

Physical activity confers neuroprotection through several partially overlapping mechanisms that complement the dietary pathways described above in Section 2.1. Amongst these, a foundational contribution is the modification of cardiometabolic risk. Exercise exerts favourable effects on blood pressure, lipid profile, insulin sensitivity, and body weight, each of which independently accelerates cognitive decline when dysregulated [58]. Building on this systemic foundation, physical activity enhances cerebrovascular function, increasing cerebral blood flow in various regions of the brain, increases production of endothelial nitric oxide synthase and supports blood–brain barrier integrity, allowing for more efficient delivery of oxygen and nutrients to the brain [59].

Beyond these vascular effects, physical activity exerts direct neurobiological actions on brain tissue. It potentially promotes hippocampal neurogenesis and strengthens synaptic connectivity chiefly through upregulation of BDNF, mediating synaptic efficacy and neuronal connectivity, and using dependent plasticity [60]. In parallel, experimental studies suggest that physical activity also facilitates glymphatic clearance of neurotoxic solutes. including amyloid-β [61], and reduces its neuronal accumulation via upregulation of the degrading enzyme neprilysin [62]. In addition, a separate pathway operates through neurotransmission, increasing the release of dopamine, norepinephrine, and serotonin across key cognitive regions, supporting attentional control, memory, and mood regulation [63].

**Figure 1:**
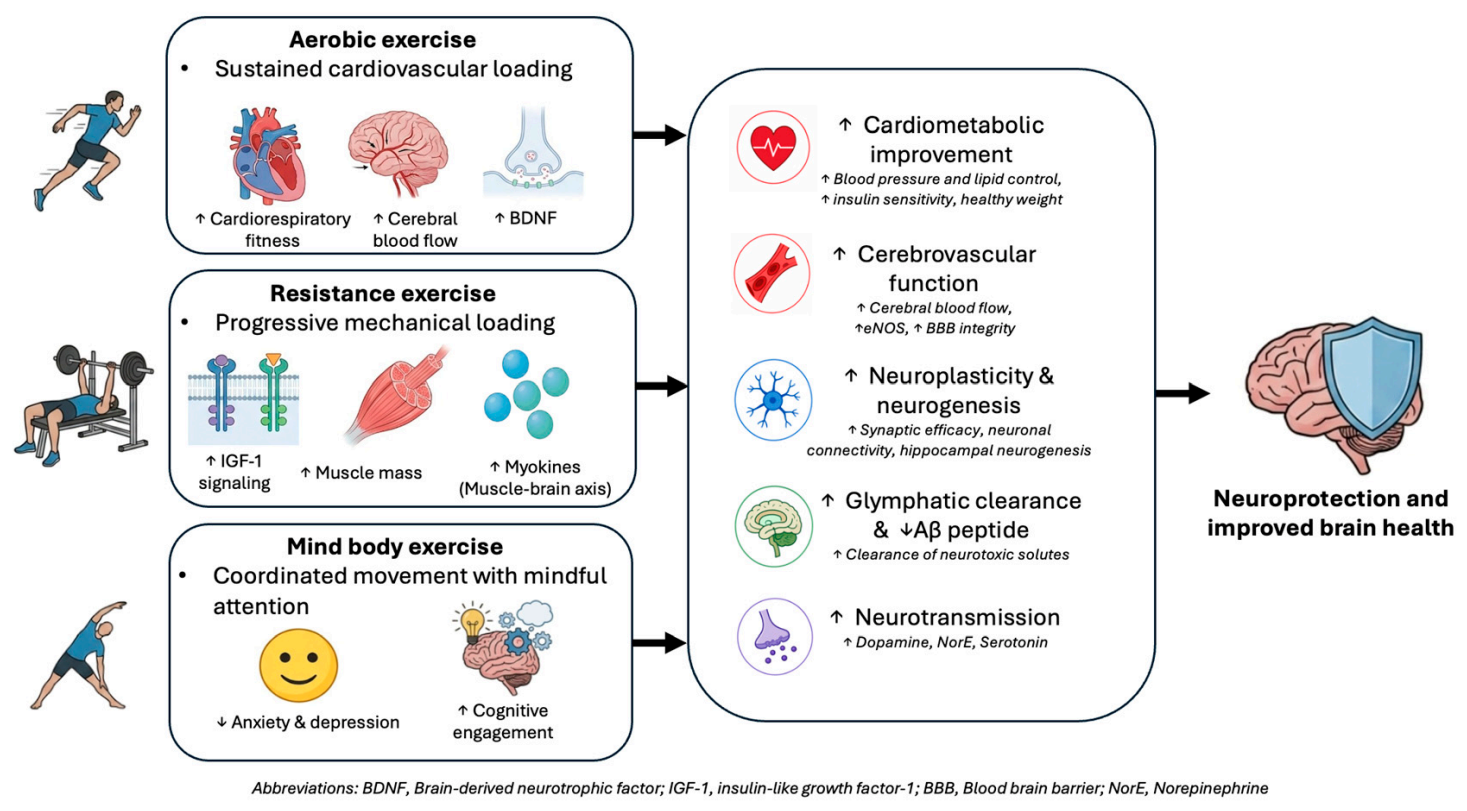
Mechanistic pathways of physical activity for cognitive outcomes.

Superimposed on these shared pathways, each exercise modality also contributes to a mechanistically distinct stimulus. Aerobic exercise is defined by sustained cardiovascular loading, driving three linked processes: improved cardiorespiratory fitness, augmented cerebral blood flow, and BDNF upregulation, with the last being directly linked to hippocampal neurogenesis and vascular neuroprotection [64]. Resistance training operates instead through insulin-like growth factor-1 (IGF-1) signalling. Resistance exercise stimulates peripheral IGF-1 secretion from liver and skeletal muscle, where circulating IGF-1 then crosses the blood–brain barrier to promote neuronal differentiation, synaptic plasticity, and angiogenesis [65], while also attenuating brain tissue loss and upregulating BDNF and vascular endothelial growth factor in support of neuronal survival and cerebrovascular integrity, as well as resistance to age-related neurodegeneration [66]. A complementary mechanism is the muscle–brain axis, in which myokines secreted by contracting skeletal muscle enter the circulation, cross the blood–brain barrier, modulate neuroinflammation, and promote hippocampal neurogenesis [67]. As the modality most reliably shown to build muscle mass and counteract sarcopenia [68], resistance training is uniquely positioned within this axis. Mind–body exercise integrates slow purposeful movement, balance, and mindful attention, deriving its benefit from the simultaneous integration of coordinated movement and sustained attentional focus rather than from cardiovascular loading or progressive resistance. Choreographed sequential movements demand skill-based learning and memorisation, activating the cingulate cortex, hippocampus, frontal lobe, and sensorimotor cortex, regions that are directly implicated in memory consolidation and executive control [69], while its meditative and relaxation components confer psychological wellbeing, reducing anxiety and depression [70], both of which are independently recognised as modifiable risk factors for cognitive decline. The proposed neurobiological mechanisms linking different physical activity modalities to cognitive outcomes are summarised in Figure 1.

### 3.2. Biomarker Evidence, Neuroimaging, and Neuropathological Studies

However, translating these mechanistic pathways into consistent clinical biomarker evidence has proven more difficult. Although animal models robustly demonstrate exercise-induced reductions in both Aβ accumulation and tau hyperphosphorylation [71], observational and interventional human studies [72,73] examining the effects of exercise on Aβ and tau biomarkers have yielded inconsistent findings. Interpretation is limited by small sample sizes, heterogeneous physical activity measurement, and biomarker endpoints that were rarely prespecified as primary outcomes. Yet, these mixed biomarker results do not necessarily negate a neuroprotective effect, but rather reflect the contribution of parallel mechanistic pathways that collectively drive cognitive protection independently of measurable amyloid or tau modification such as vascular mechanisms.

At the structural level, evidence is more encouraging [74]. A large neuroimaging study of 10,125 individuals [75] found that moderate to vigorous physical activity was associated with greater volumes of total grey matter, the hippocampus, frontal cortex, parietal lobe, and occipital lobe, while longitudinal cohort evidence [76] suggests that these structural benefits accumulate across the lifespan. Individual interventional studies have also shown that aerobic exercise can structurally reverse age-related brain atrophy. One of the landmark studies by Erickson et al. demonstrated that one year of aerobic training increased the anterior hippocampal volume by approximately 2%, effectively adding 1 to 2 years of hippocampal volume to older adults who would otherwise have experienced continued age-related decline [77]. However, a later meta-analysis [78] on 14 interventional studies (n = 737) showed that aerobic exercises had no significant effect on the total hippocampal volume, though a sub-analysis of nine studies (n = 501) patients showed that there was a small positive effect on the left hippocampal volume. Beyond the hippocampus, aerobic exercise has also been found to increase grey and white matter volume in the frontal lobes over 6 months, with these structural benefits appearing most pronounced in older age groups [79]. This is also further supported by a large cross-sectional study of the UK Biobank (n = 16,725) [80] where it showed that greater physical activity, as determined over a 7 day accelerometer measurement, had an association with total brain volume, grey matter, white matter, and total hippocampal volume, while being inversely associated with white matter hyperintensity volume.

Meanwhile, progressive resistance training has also been shown to attenuate hippocampal atrophy in older adults, protect white matter integrity, increase grey matter cortical thickness, and produce functional changes in the frontal lobe, all of which correspond to the executive function and memory domains improved by resistance training in a systematic review of interventional and cross-sectional studies [65].

However, translating these associations into trial evidence across mixed exercise modalities has proven more difficult. A 2021 systematic review [81] of 19 RCTs using various types of exercise modalities found the neuroimaging findings to be mixed and highly heterogeneous, with no consistent protective effect on grey matter volume detectable across trials. However, these null RCT findings must be interpreted with caution, as the included trials were all short-term (≤12 months) and underpowered given that neuroimaging was not a primary outcome, and a subset examined neurological conditions unrelated to neurodegeneration, all of which substantially limit the reliability of the negative conclusion. Adequately powered, long-duration trials with amyloid and tau biomarkers, and neuroimaging as prespecified primary endpoints, are therefore needed before definitive conclusions can be drawn.

### 3.3. Key Clinical Evidence and Prescription Parameters: Observational and Interventional Studies

Physical activity is among the most frequently recommended interventions in clinical practice, yet among the least precisely prescribed. Where the MIND diet offers clinicians a discrete 15-item list, exercise recommendations are often reduced to generic encouragement, obscuring the fact that modality, intensity, and frequency each carry distinct neurobiological consequences. Meaningful appraisal therefore requires that the modalities and their prescription parameters be cross-examined with the evidence.

Across modalities, exercise interventions have generally demonstrated cognitive benefit, even as their relationships with biomarkers, neuroimaging, and neuropathological measures remain inconsistent. A systematic review and meta-analysis of nine studies reported that aerobic exercise produces significant improvements in memory and executive function [82]. Meta-analytic data from Landrigan et al. [83] and Coelho Junior et al. [84] collectively demonstrate that resistance training produces significant improvements in global cognitive function, executive function, and memory across both cognitively healthy older adults and those with mild cognitive impairment, with pooled effect sizes in the moderate range. Meta-analytic evidence across 45 studies involving 4602 cognitively intact and mildly cognitively impaired participants [69] indicates that mind–body exercise produces a moderate effect on visuospatial construction and small but statistically significant effects on verbal memory, processing speed, working memory, and executive function, with MCI populations consistently deriving greater cognitive benefit than cognitively healthy older adults in the visuospatial construction and executive function domains. A complementary network meta-analysis of 58 RCTs [85] further identified mind–body exercise as the most efficacious modality for improvement of overall cognitive function, especially in domains of executive function and working memory among cognitively unimpaired older adults, outperforming aerobic exercise and resistance exercises on these specific domains.

However, what is not so clear about this body of evidence presented so far is the prescription of exercise required to achieve cognitive improvement. Exercise prescription is conventionally specified through the FITT framework: frequency, intensity, time, and type [86].

Current international guidance provides a reference standard for the time and frequency components of prescription. Both the World Health Organization [87] and the U.S. Physical Activity Guidelines Advisory Committee [88] recommend that older adults undertake at least 150 to 300 min of moderate-intensity aerobic activity per week, together with muscle-strengthening activity of at least moderate intensity on two or more days per week, and, for those with reduced mobility, multicomponent activity emphasising balance and functional training on three or more days per week. Unfortunately, there is no harmonised intensity metric spanning across all three modalities. While such a metric would simplify prescription, these modalities act through substantively different mechanisms, and a common intensity scale would confer an appearance of comparability that the biology does not support.

Aerobic exercise intensity can be quantified as a relative to maximum heart rate (%MHR). No specific aerobic modality has shown to be superior for cognitive outcomes due to the lack of evidence. Hence, the guiding principle is to achieve a sufficient cardiovascular stimulus, typically expressed as a target percentage of the maximum heart rate, while allowing individuals to self-select the activity most compatible with their preferences, fitness levels, and comorbidities, a patient-centred approach that will likely optimise long-term adherence.

Evidence supports moderate-intensity aerobic exercise, performed at 50–70% MHR, as the best-evidenced and most universally applicable prescription for cognitive protection in older adults. Studies exploring the dose–response relationship between exercise intensity and cognitive outcomes revealed that moderate-intensity protocols have the most robust and consistent evidence base, while high-intensity aerobic exercise (70–85% MHR) has produced heterogeneous results across trials, and low-intensity aerobic exercise has demonstrated mixed and less convincing cognitive efficacy [89]. This picture is not unchallenged. In EXERT, the largest trial to test it directly, 18 months of aerobic training at 70–80% heart rate reserve, did not outperform low-intensity stretching and balance work in adults with amenestic mild cognitive impairment [90]. Neither group declined cognitively over 12 months, and 12-month hippocampal volume loss was low in both at 0.51% [91]. Both arms were active, and this bears on the relative value of intensity rather than on whether exercise confers benefit at all. From a practical standpoint, moderate-intensity exercise represents the most clinically pragmatic threshold for this population where many older adults present with sedentary lifestyles, multiple comorbidities, and reduced exercise tolerance, rendering high-intensity protocols poorly tolerated and poorly adhered to, while low-intensity activity may fall below the stimulus required to drive meaningful neurobiological adaptation. Importantly, exercise intensity should not be evaluated purely through the lens of cognitive outcomes in isolation. The broader cardiometabolic benefits of sustained moderate-intensity aerobic exercise, including improvements in blood pressure, lipid profiles, insulin sensitivity, and cardiovascular fitness, have been linked to lower dementia risk [92], and represent an important co-primary goal of any exercise prescription. Volume, unlike intensity, can be placed on a common scale. Within aerobic exercise, the metabolic equivalent of task minutes per week (METs-min/week) standardises dosing by multiplying intensity by duration across the weekly training load. Based on a Bayesian model-based network meta-analysis of 44 RCTs [93] involving 4793 older adults, the estimated minimum exercise dose associated with a clinically meaningful improvement in global cognition was 724 METs-min per week, which was slightly above the WHO-recommended lower bound of 600 METs-min per week. Cognitive benefits increased markedly, up to approximately 1200 METs-min per week, beyond which additional volume yielded diminishing returns.

For resistance exercises, like aerobic exercise, current evidence does not support the superiority of any specific resistance modality either, which have all demonstrated broadly comparable efficacy for cognitive outcomes [83]. Rather than prescribing a specific apparatus, the guiding principle is progressive overload, which involves systematically increasing resistance over time to sustain an adequate neuromuscular and neurotrophic stimulus. This principle should guide prescription regardless of modality, and individuals should be encouraged to self-select the format most compatible with their physical capacity, joint health, and personal preference.

Regarding specific prescription parameters, intensity can be seen as a percentage of one repetition maximum (%1RM). NSCA guidelines [94] recommend 2–3 sets of 1–2 multi-joint exercises per major muscle group, achieving intensities of 70–85% of one repetition maximum (1RM), 2–3 times per week. Current evidence suggests that resistance exercise performed at approximately 60–80% of 1RM may confer cognitive benefits, with comparable effects observed across moderate and higher intensities within this range [66]. However, the optimal intensity remains uncertain given the heterogeneity across studies, allowing for individualisation according to physical capacity and tolerance. Similarly, programmes of at least 16 weeks in duration, with a minimum frequency of two sessions per week, represent the threshold most consistently associated with cognitive benefits in the literature [84], although these parameters should not be regarded as definitive minimum thresholds. These findings broadly align with WHO recommendations for twice-weekly moderate- or greater-intensity muscle-strengthening activity in older adults, whose cognitive protective rationale is now substantially reinforced by the mechanistic and clinical evidence reviewed here.

Unfortunately, when it comes to mind–body exercise, no equivalent consensus metric exists. Perhaps this is better explained by the fact that its cognitive stimulus derives from skill-based learning and sustained attentional demand rather than from cardiovascular or mechanical loading. Among the exercise subtypes, Tai Chi, Yoga, and Qigong demonstrate meaningfully greater effects [69] on visuospatial construction compared to meditation alone, a distinction likely attributable to the fact that these modalities impose considerable spatial processing demands through proprioceptive engagement and goal-directed upper body movements that activate the parietal lobe, whereas meditation is a stationary behaviour that does not generate equivalent neuromotor stimulation. Despite the absence of any harmonised intensity metric or clear guidance on optimal modality, mind–body exercise has a clear clinical utility. Its low impact and accessible nature make it particularly well suited for individuals with limited exercise tolerance, physical frailty, high fall risk, or substantial comorbidities, groups for whom conventional aerobic or resistance training may be impractical or contraindicated. It should therefore be positioned not as a substitute, but as a complementary component within a comprehensive, multimodal exercise strategy for cognitive preservation.

## 4. Multidomain Interventions

Although MIND dietary modification and physical activity each have substantial independent evidence supporting their role in cognitive outcomes and converge on similar core neurobiological substrates, direct evidence evaluating their synergistic effects remains limited. Studies examining diet and exercise together as a two-component intervention are few and highly heterogenous [95] with respect to study population and intervention design, making it difficult to draw firm conclusions regarding their combined efficacy. Evidence specifically evaluating the MIND diet together with structured physical activity and their interaction effects is further limited [96,97,98,99]. The two RCTs [96,97] examined specific exercise modalities and selected populations, including Baduanjin exercise and obese postmenopausal women, limiting the generalisability of their findings. Meanwhile, retrospective single-cohort studies [98,99] have not demonstrated consistent evidence of synergistic cognitive benefits from combining MIND diet adherence with physical activity. However, the limited number of studies and their methodological constraints preclude definitive conclusions regarding potential synergistic effects.

Cognitive impairment is a multifactorial condition, and the Lancet Commission has identified numerous modifiable risk factors spanning vascular, metabolic, behavioural, and psychosocial domains, suggesting that effective prevention is unlikely to depend on the modification of any two factors alone. Hence, it is also clinically relevant to examine MIND or MIND-aligned dietary modification and physical activity as foundational components within the broader multidomain interventions trials that provide an important evidence base for understanding how these two lifestyle cornerstones contribute to cognitive preservation.

The first major proof of concept for this approach was provided by the Finnish Geriatric Intervention Study to Prevent Cognitive Impairment and Disability (FINGER) trial [9]. FINGER randomised 1260 adults aged 60–77 years with elevated dementia risk to a two-year multidomain intervention comprising dietary modification, aerobic and resistance exercise, cognitive training, and vascular risk monitoring, compared with standard health advice. The dietary intervention was based on the Finnish Nutrition Recommendations [100] rather than the MIND diet itself. Nevertheless, there is substantial conceptual and compositional overlap between the two approaches, including an emphasis on vegetables, fruits and berries, whole grains, fish, and unsaturated fats, together with restriction of saturated fats, excess sugar, and other nutritionally adverse foods. FINGER therefore provides an important proof of concept that dietary modification broadly consistent with MIND-aligned principles, together with structured physical activity, can be successfully embedded within a comprehensive dementia-prevention programme. Over two years, the intervention group demonstrated significantly greater improvement in the neuropsychological test battery composite score than controls, which was equivalent to 25% higher in the interventional group than in control group. In addition, the intervention’s adherence rate across all four intervention components was high, with 72% of participants completing all four domains, thus reinforcing the real-world feasibility of intensive multidomain lifestyle intervention.

This model subsequently formed the basis of the Worldwide FINGERS (WW-FINGERS) network and was extended more directly to the MIND diet in the US Study to Protect Brain Health Through Lifestyle Intervention to Reduce Risk (US POINTER) [10]. US POINTER is particularly relevant to the present review because the structured intervention explicitly incorporated the MIND diet alongside aerobic, resistance, and flexibility exercise, cognitive engagement, and health coaching. Among 2111 cognitively unimpaired older adults at increased risk of cognitive decline, global cognitive performance improved in both study groups over two years, but improved significantly faster in those receiving the structured multidomain intervention. The effect was greater among participants with lower baseline cognitive performance, while mean team-meeting attendance remained high at 91% in the structured intervention group.

More recently, LatAm-FINGERS extended this framework across 11 Latin American countries, demonstrating that the multidomain model could be adapted successfully across markedly different cultural, socioeconomic, and dietary settings [11]. Among 1065 older adults with elevated dementia risk and suboptimal cognition, the structured lifestyle intervention which included regular MIND diet intervention counselling and a highly structured exercise programme involving aerobic, resistance, coordination and balance training produced a significantly greater improvement in global cognition over two years than the flexible comparator. In addition, unlike the earlier trials, exercise components were prescribed explicitly with four supervised sessions weekly, comprising 35 min of aerobic exercise, and twice-weekly 15 min resistance training and twice-weekly 15 min coordination and balance training, at 55–65% of maximum heart rate progressing to 70–80% after six months—a transferable FITT specification. Additionally, feasibility was also assessed prospectively as a co-primary outcome using RE-AIM measures, an approach well suited to complex behavioural interventions where delivery is as uncertain as efficacy [101]. The mean session attendance and the two-year retention rate were high, with a lower dropout rate in the systematic lifestyle intervention arm (15.2% vs. 20.2%).

It is, however, also important to note that the broader multidomain literature has not been uniformly positive. Earlier trials such as preDIVA [102] and MAPT [103], as well as subsequent WW-FINGERS studies including J-MINT [12] and AgeWell.de [13], did not demonstrate significant cognitive benefit in their primary analyses. However, these studies should not be considered directly equivalent to tests of the intervention strategy discussed here. None incorporated the MIND diet specifically, and there were substantial methodological differences in the nature and intensity of dietary counselling, exercise delivery, degree of supervision, study population, and comparator conditions. Their neutral findings therefore highlight the considerable heterogeneity inherent within multidomain intervention research rather than providing direct evidence against the combination of MIND dietary principles and structured physical activity.

Taken together, the most informative positive multidomain trials suggest an evolving continuum of evidence: FINGER established the initial proof of concept using a dietary pattern that substantially overlaps with MIND principles with physical activity as cornerstones; US POINTER subsequently demonstrated cognitive benefit in a structured intervention explicitly incorporating the MIND diet; and LatAm-FINGERS showed that the overall framework can be successfully adapted across diverse populations. However, the magnitude of benefit is generally modest and improvements in cognitive test scores do not necessarily establish reductions in dementia incidence. Importantly, these trials included additional components such as cognitive training and vascular risk management, making it impossible to isolate the individual or synergistic contributions of the MIND diet and physical activity. Rather, they support conceptualising dietary modification and physical activity as two complementary crucial lifestyle modifications within a broader, multifactorial strategy for cognitive improvement. The key multidomain intervention trials examining cognitive outcomes, including their intervention components and principal findings, are summarised in Table 3.

## 5. Discussion and Conclusions

Across cohorts and trials, the relationship between baseline risk and observed benefit appears nonlinear rather than simply graded. At the low-risk end of the spectrum, intervention effects may be difficult to detect because cognitive decline and event rates are modest over the duration of follow-up, resulting in floor effects and limited statistical power. Conversely, at the high-risk end, where more established neuropathological changes may already be present, the potential for modification may also be attenuated. This raises the possibility that lifestyle interventions of the MIND diet and physical activity confer the greatest benefit among individuals at an intermediate stage of risk, those who are cognitively vulnerable but have not yet developed substantial irreversible pathology. However, a nonlinear relationship has not been formally established, and differences in intervention intensity, adherence, follow-up duration, comparator conditions, and cognitive outcome measures may also explain the variation across studies. Further prospective studies are needed to determine whether baseline risk modifies intervention efficacy and to identify populations most likely to benefit.

A practical strength of the MIND diet is its tiered adherence model. Even moderate adherence is associated with meaningful risk reduction, making it a realistic, prescribable framework for a broad patient population. Its emphasis on accessible whole foods aligns with existing nutritional guidelines and avoids the limitations of supplement-mediated nutrient delivery. For physical activity, the different modalities benefit cognition through partially distinct mechanisms, which support multimodality rather than single-modality prescription, although modality-specific trials remain heterogeneous in intensity, duration, and outcome measurement. Multidomain interventions that simultaneously target MIND dietary quality, physical activity, cognitive training, and vascular risk management currently carry the most replicable trial evidence. FINGER, US POINTER, and LatAm-FINGERS each demonstrated statistically significant improvements in composite cognitive scores among at-risk older adults, and each delivered its intervention in a structured, facilitated format with good adherence rather than as generic behavioural advice. Although these findings provide encouraging evidence, they are derived from a limited number of trials and do not establish the independent contributions of individual intervention components. Adherence appears to matter a great deal. The trials reporting benefit are generally those with high session attendance under supervision, which raises the possibility that simple advice may yield little benefit in clinical practice, and that patients may instead need referral to a structured programme designed to support adherence. Nevertheless, structured programmes require additional resources and supervision, while potential exercise-related adverse events should be considered, particularly in frail or multimorbid older adults.

An important clinical context for these findings is the expanding but substantially constrained availability of anti-amyloid therapies (AATs). The approvals of lecanemab and donanemab are genuine advances, demonstrating modest slowing of cognitive decline in early Alzheimer’s disease. However, eligibility requires confirmed amyloid pathology via PET or cerebrospinal fluid testing, is restricted to MCI or mild dementia, and carries a high annual drug cost. Amyloid-related imaging abnormalities also occur in 20–30% of participants across trials [104], necessitating serial MRI and specialist oversight. Although AAT primarily targets patients with mild cognitive impairment to early symptomatic Alzheimer’s disease, lifestyle interventions have broader applicability across populations at risk of cognitive decline in general. Rather than viewing lifestyle interventions and AATs as competing strategies, a complementary paradigm is emerging in which multidomain lifestyle modification serves as a relatively accessible, low-risk foundation for cognitive preservation, addressing multiple pathological pathways simultaneously while remaining especially relevant for individuals in whom AAT is clinically unsuitable or practically or economically inaccessible.

Several limitations temper these conclusions. First, most of the MIND diet evidence is observational, and such studies are open to healthy user bias, residual socioeconomic confounding, and reverse causation, since prodromal neurodegeneration can alter food preference and physical activity years before diagnosis. Exposure measurement is a further limitation throughout: MIND scores and physical activity are largely self-reported and subject to misclassification, and cognitive outcomes are captured with instruments that are not interchangeable across studies. RCT evidence on the MIND diet alone remains sparse, and the one landmark trial was negative. Second, the physical activity literature is uneven across levels of evidence. Biomarker and structural imaging findings are heterogeneous and often derive from trials in which these were secondary endpoints, whereas the interventional evidence for cognitive outcomes is somewhat more consistent, though still variable in modality, intensity, and how outcomes were measured. Third, most of the evidence presented here used cognitive test performance as an outcome rather than a clinical disease state such as dementia. Improvement in cognitive scores may not translate into reduced risk of MCI or dementia, which is the outcome that matters most to patients and clinicians.

Priority future research directions should include, firstly, the minimum duration of interventions. Adequately powered, long-duration MIND diet trials are needed to establish how long adherence must be sustained before a cognitive effect emerges, ideally with plasma AD biomarkers and structural imaging as prespecified endpoints rather than secondary ones. The second is dosing. Head-to-head exercise trials are needed to determine the minimum prescription that yields cognitive benefit, since current guidance specifies frequency and duration but not the threshold at which benefit begins. The third is attribution. Mechanistic and factorial studies are needed to separate the individual contributions of diet and exercise from any synergy between them, which no trial to date has been designed to do. The fourth is delivery. Implementation research is needed to develop scalable, culturally adaptable models across diverse populations, while also understanding the cost-effectiveness of such structured interventions.

The MIND diet and physical activity represent two promising, modifiable lifestyle strategies for cognitive preservation and dementia risk reduction, supported by a growing body of evidence. Their mechanisms of action are potentially complementary, with experimental evidence suggesting that the MIND diet constituents may influence amyloid metabolism, neuroinflammation, and vascular health, while physical activity may support cerebrovascular function, neuroplasticity, and glymphatic clearance. However, the extent to which these mechanisms mediate cognitive benefits in humans remains uncertain. The convergence of mechanistic, biomarker, observational, and emerging trial data provides a supporting rationale for shifting from siloed lifestyle recommendations toward an integrated, multimodal strategy that combines MIND dietary optimisation with tailored exercise prescription. This approach is accessible, safe, and broadly applicable, and generally well tolerated when supervised, addressing not only cognitive trajectories but overall health and function across the lifespan, and offering an essential and equitable counterpart to pharmacological disease modification in the evolving dementia-prevention landscape.

## Figures and Tables

**Table 2:** Summary of key clinical studies on the MIND diet and cognitive outcomes.

Study	Design	Population	Key Findings
Morris et al., 2015 [14]	Prospective MAP cohort; 10 years follow-up	n = 960; Chicago, USA retirement community; mean age 81.4 years; free of dementia	Top and middle MIND tertile protective against AD compared to lowest tertile, with a 7.5-year difference in cognitive age between the extremes; association also present for cognitively unimpaired participants
Morris et al., 2015 [6]	Prospective MAP cohort; 4.5 years follow-up	n = 923; Chicago, USA retirement community; age range 58–98 years; free of dementia	Lower AD incidence in middle (HR 0.65, 95% CI 0.44–0.98) and highest MIND adherence tertiles (HR 0.47, 95% CI 0.29–0.76), versus lowest tertile
Berendsen et al., 2018 [54]	Prospective NHS cohort; 6 years follow-up	n = 16,058; American women; mean age 74.3 years; free of stroke	Higher MIND adherence has better verbal memory but no effect on global cognition or TICS score. Restricting butter/margarine and pastries drove the verbal memory association.
Hosking et al., 2019 [7]	Prospective PATH Through Life cohort; 12 years follow-up	n = 1220 Australian adults; cognitively unimpaired; mean age 62 years	MIND, not MeDi, was associated with reduced 12-yr cognitive impairment odds, with odds reducing by 19% with every 1-point increase in MIND score
Melo van Lent et al., 2021 [56]	Prospective FHS cohort; 6.6 years mean follow-up	n = 2092 American adults; mean age 61 years; free of dementia and stroke	Higher MIND adherence associated cross-sectionally with better cognitive scores and larger total brain volume at baseline; however, there was no association with cognitive decline over follow-up
Huang et al., 2023 [57]	Prospective CHNS cohort; 3 years median follow-up	n = 4066; Chinese; mean age 62.2 years	Higher modified MIND score associated with better global cognitive function; each 3-pt MIND score increment ≈ 1 yr younger cognitive age
Barnes et al., 2023 [8]	RCT; 1:1 MIND diet vs. control diet (both with caloric restriction); 3 years intervention	n = 604; American adults with family history of dementia, BMI ≥ 25 and suboptimal MIND diet ≤ 8,	Both groups improved with no significant between-group difference in global cognitive scores; changes in MRI associated with cognitive function are similar in both groups; both groups had similar weight loss
Sager et al., 2024 [55]	Prospective sub-study within DO-HEALTH RCT; 3 years follow-up	n = 2028 community generally healthy adults, MMSE ≥ 24; mean age 74.9 years; mean BMI 26.3	No association between MIND diet adherence and cognitive decline, likely floor effect in low-risk cognitively unimpaired population

Abbreviations: CHNS = China Health and Nutritional survey; HR = Hazard ratio; MAP = Memory and Aging Project; MeDi = Mediterranean diet; NHS = Nurses’ Health Study; RCT = randomised controlled trial; and TICS = telephone interview for cognitive status.

**Table 3:** Summary of key clinical studies on multidomain interventions and cognitive outcomes.

Study	Design	Population	Key Findings
MIND or MIND-aligned diet and physical activity as part of a multidomain intervention			
FINGER, 2015 [9]	Supervised multidomain vs. standard health advice; 2 years follow-up	n = 1260 Finnish; Age 60–77; average to slightly lower than expected cognition for age; CAIDE ≥ 6	Intervention group demonstrated better performance on a global cognitive test score with a between-group Z score difference of 0.022 SD/year; adherence was found to be 72%
US POINTER, 2025 [10]	Supervised multidomain intervention vs. self-guided lifestyle intervention; 2 years follow-up	n = 2111 Americans; higher risk of cognitive decline based on specific physical activity level, diet, family history, cardiometabolic risk, specific ethnicity, age, sex	Improved global cognitive test score for multidomain intervention group with a difference of 0.029 SD/year; adherence was at 91% (measured using team-meeting attendance)
LatAM-FINGERS, 2026 [11]	Supervised multidomain intervention vs. flexible lifestyle intervention; 2 years follow-up	n = 1065 Latin Americans; age 60–77; average to slightly lower than expected cognition for age; CAIDE ≥ 6	Improved global cognitive test score for multidomain intervention group with a between-group difference of 0.11 SD/year; adherence was at 71%
Other landmark multidomain intervention trials that do not include MIND diet specifically			
PreDIVA, 2016 [102]	Nurse-led, multidomain intervention vs. usual care; 6 years follow-up	n = 3526 Dutch; age 70–78; without dementia	No significant difference in the primary analysis of dementia diagnosis after 6 years; however, per-protocol analysis hazard ratio moved toward benefit (HR 0.78, 95% CI 0.58–1.04); participants with untreated hypertension at baseline who were adherent had significantly reduced dementia incidence (HR 0.54, 95% CI 0.32–0.92)
MAPT, 2017 [103]	Directly unsupervised multidomain intervention with or without omega-3 supplementation, or to omega-3 or placebo alone (1:1:1:1); 3 years follow-up	n = 1680 French and Monegasque; Age ≥ 70 without dementia with MMSE ≥ 24 with either spontaneous memory complaint or limitation in iADL or slow gait speed	No difference in cognitive test scores between-group; adherence was found to be 50+% across 3 years; physical activity was unchanged in intervention group; post hoc analysis of participants allocated to multidomain intervention against those who were not showed a significant difference in the primary outcome
Agewell.de, 2023 [13]	Unsupervised multidomain intervention vs. usual care; 2 years follow-up	n = 1030 German; Age 60–77; CAIDE ≥ 9	No difference in global cognitive test scores between-group; intervention intensity and adherence were identified as potential contributors to the neutral findings
J-MINT, 2024 [12]	Supervised multidomain intervention vs. usual care; 18 months follow-up	n = 531 Japanese; age 65–85; Known MCI	No difference in global cognitive test scores between-group; a prespecified subgroup analysis showed that participants attending ≥70% of group exercise sessions had a significant improvement in primary outcome relative to both non-adherent participants and control

## Data Availability

No new data were created or analyzed in this study. Data sharing is not applicable to this article.
